# 2-{(*E*)-[(2-Methyl-3-nitro­phen­yl)imino]­meth­yl}-4-nitro­phenol

**DOI:** 10.1107/S1600536813015407

**Published:** 2013-06-12

**Authors:** Hasan Tanak, Ferhat Toğurman, Sedanur Kalecik, Necmi Dege, Metin Yavuz

**Affiliations:** a Department of Physics, Faculty of Arts & Science, Amasya University, Ipekkoy-Amasya, Turkey; b Department of Physics, Faculty of Arts & Science, Ondokuz Mayıs University, TR-55139, Kurupelit-Samsun, Turkey; c Faculty of Technology, Amasya University, TR-05100 Amasya, Turkey

## Abstract

The title compound, C_14_H_11_N_3_O_5_, is a Schiff base that adopts the enol–imine tautomeric form in the solid state. The dihedral angle between the aromatic rings is 37.4 (3)° and the dihedral angles between the nitro groups and their attached rings are 4.0 (6) and 46.2 (8)°. The mol­ecular structure is stabilized by an intra­molecular O—H⋯N hydrogen bond, which generates an *S*(6) ring motif. In the crystal, molecules are linked by C—H⋯O interactions, forming a two-dimensional network parallel to the *bc* plane.

## Related literature

For the biological properties of Schiff bases, see: Aydoğan *et al.* (2001 ▶); Taggi *et al.* (2002 ▶); Barton & Ollis (1979 ▶); Layer (1963 ▶); Ingold (1969 ▶); Cohen *et al.* (1964 ▶); Moustakali-Mavridis *et al.* (1978 ▶). For tautomeric forms of Schiff base compounds, see: Tanak *et al.* (2010 ▶). For hydrogen-bond motifs, see: Bernstein *et al.* (1995 ▶). For a related structure, see: Tanak (2011 ▶).
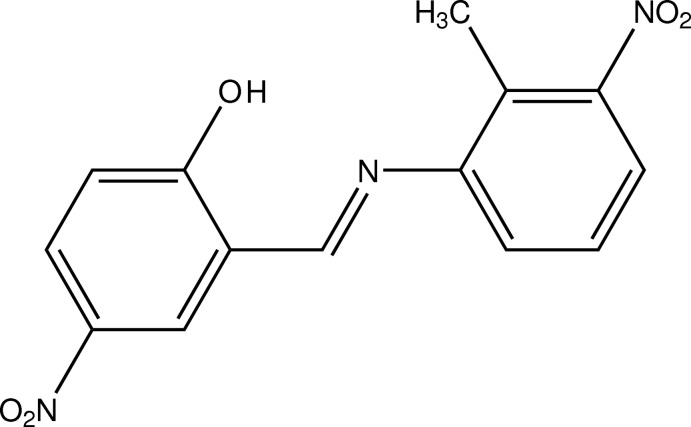


## Experimental

### 

#### Crystal data

C_14_H_11_N_3_O_5_
*M* *_r_* = 301.26
Monoclinic, 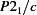

*a* = 3.754 (5) Å
*b* = 15.696 (5) Å
*c* = 23.149 (5) Å
β = 93.491 (5)°
*V* = 1361.5 (19) Å^3^
*Z* = 4
Mo *K*α radiation
μ = 0.11 mm^−1^
*T* = 296 K
0.46 × 0.20 × 0.05 mm

#### Data collection

Stoe IPDS II diffractometer
Absorption correction: integration (*X-RED32*; Stoe & Cie, 2002 ▶) *T*_min_ = 0.600, *T*_max_ = 0.976
7609 measured reflections
2538 independent reflections
923 reflections with *I* > 2σ(*I*)
*R*_int_ = 0.181

#### Refinement

*R*[*F* ^2^ > 2σ(*F* ^2^)] = 0.095
*wR*(*F* ^2^) = 0.229
*S* = 0.97
2538 reflections
199 parameters
H-atom parameters constrained
Δρ_max_ = 0.23 e Å^−3^
Δρ_min_ = −0.25 e Å^−3^

### 

Data collection: *X-AREA* (Stoe & Cie, 2002 ▶); cell refinement: *X-AREA*; data reduction: *X-RED32* (Stoe & Cie, 2002 ▶); program(s) used to solve structure: *SHELXS97* (Sheldrick, 2008 ▶); program(s) used to refine structure: *SHELXL97* (Sheldrick, 2008 ▶); molecular graphics: *ORTEP-3 for Windows* (Farrugia, 2012 ▶); software used to prepare material for publication: *WinGX* (Farrugia, 2012 ▶).

## Supplementary Material

Crystal structure: contains datablock(s) I. DOI: 10.1107/S1600536813015407/bt6911sup1.cif

Structure factors: contains datablock(s) I. DOI: 10.1107/S1600536813015407/bt6911Isup2.hkl

Supplementary material file. DOI: 10.1107/S1600536813015407/bt6911Isup3.cml

Additional supplementary materials:  crystallographic information; 3D view; checkCIF report

## Figures and Tables

**Table 1:** Hydrogen-bond geometry (Å, °)

*D*—H⋯*A*	*D*—H	H⋯*A*	*D*⋯*A*	*D*—H⋯*A*
O3—H2⋯N2	0.82	1.85	2.589 (8)	149
C6—H6⋯O2^i^	0.93	2.50	3.333 (9)	149
C4—H4⋯O4^ii^	0.93	2.59	3.274 (9)	131

Symmetry codes: (i) 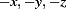; (ii) 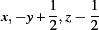.

**Fig. 1.**
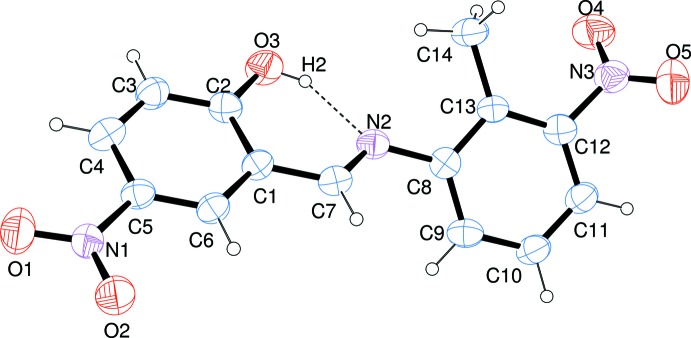
The molecular structure of the title compound, showing the atom-numbering scheme and 30% probability diplacement ellipsoids. The intramolecular hydrogen bond is shown as a dashed line.

**Fig. 2.**
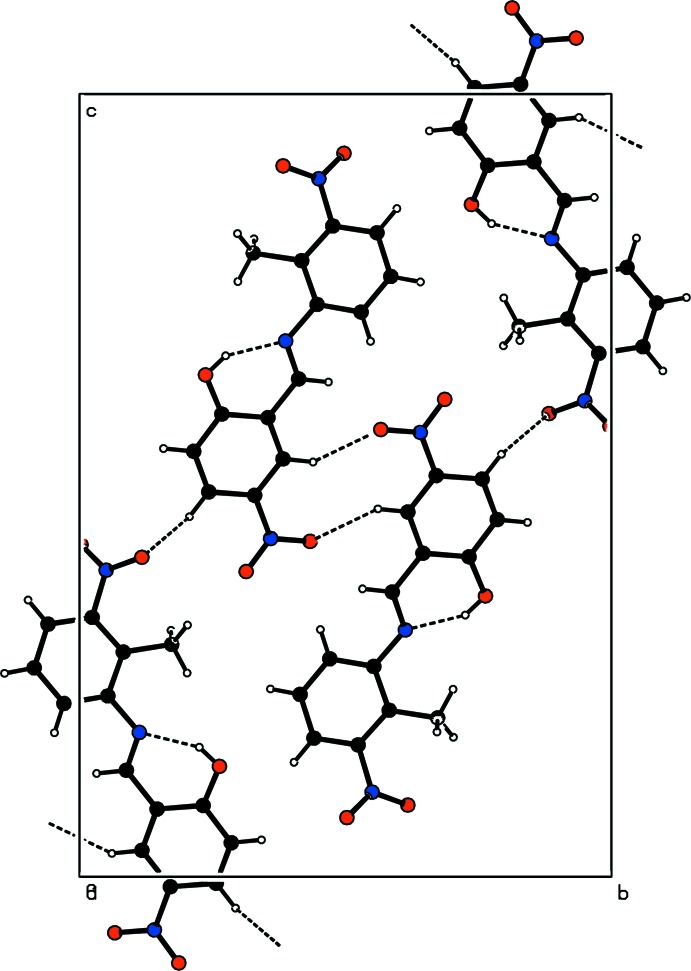
The crystal packing of the title compound. Hydrogen bonds are shown as dashed lines.

## supplementary crystallographic information

### Comment

Schiff bases, *i.e.*, compounds having a double C=N bond, are used as starting materials in the synthesis of important drugs, such as antibiotics and antiallergic, antiphlogistic, and antitumor substances (Barton *et al.*, 1979; Layer, 1963; Ingold 1969). On the industrial scale, they have a wide range of applications, such as dyes and pigments (Taggi *et al.*, 2002). Schiff bases have also been employed as ligands for the complexation of metal ions (Aydoğan *et al.*, 2001). There are two characteristic properties of Schiff bases, *viz*. Photochromism and thermochromism (Cohen *et al.*, 1964). In general, Schiff bases display two possible tautomeric forms, the enol-imine (OH) and the keto-amine (NH) forms. Depending on the tautomers, two types of intramolecular hydrogen bonds are observed in Schiff bases: O—H···N in enol-imine and N—H···O in keto-amine tautomers (Tanak *et al.*, 2010).

In the title crystal structure (Fig. 1), the molecules of the title compound are not planar. The dihedral angle between the aromatic ring systems is 37.4 (3)°. The imino group is coplanar with the hydroxyphenyl ring as it can be shown by the C2—C1—C7—N2 torsion angle [-1.6 (8)°]. The C—O and C=N bond lengths confirm the enol-imine form of the title compound. The length of the C7=N2 double bond is 1.269 (7) Å It is consistent with the related structure (Tanak, 2011). It is also known that Schiff bases may exhibit thermochromism depending on the planarity or non-planarity of the molecule, respectively (Moustakali-Mavridis *et al.*, 1978).

The molecular structure is stabilized by an intramolecular hydrogen bond. The phenol H atom forms a strong intramolecular hydrogen bond with the imine N atom (Fig. 1) generating an S(6) ring motif (Bernstein *et al.*, 1995). In the crystal structure, molecules are linked together by intermolecular C—H···O interactions (Fig. 2).

### Experimental

2-hydroxy-5-nitrobenzaldehyde (0.0138 g, 0.0822 mmol) was added to a solution of 2-methyl-3-nitroaniline (0.0125 g, 0.0822 mmol) in ethanol (100 ml). The reaction mixture was stirred for 24 h under reflux. Single crystals suitable for X-ray analysis were obtained from ethyl alcohol by slow evaporation (yield 52%; m.p.485–487 K).

### Refinement

All H atoms were positioned geometrically and treated using a riding model, fixing the bond lengths at 0.82 Å for OH, at 0.93 Å for aromatic CH, at 0.96 Å for CH_3_. The displacement parameters of the H atoms were constrained as *U*_iso_(H)= 1.2*U*_eq_(1.5*U*_eq_for methyl) of the parent atom.

### Crystal data

**Table:**

C_14_H_11_N_3_O_5_	*F*(000) = 624
*M**_r_* = 301.26	*D*_x_ = 1.470 Mg m^−^^3^
Monoclinic, *P*2_1_/*c*	Mo *K*α radiation, λ = 0.71073 Å
Hall symbol: -P 2ybc	Cell parameters from 6502 reflections
*a* = 3.754 (5) Å	θ = 1.6–26.2°
*b* = 15.696 (5) Å	µ = 0.11 mm^−^^1^
*c* = 23.149 (5) Å	*T* = 296 K
β = 93.491 (5)°	Stick, yellow
*V* = 1361.5 (19) Å^3^	0.46 × 0.20 × 0.05 mm
*Z* = 4	

### Data collection

**Table:**

Stoe IPDS II diffractometer	2538 independent reflections
Radiation source: fine-focus sealed tube	923 reflections with *I* > 2σ(*I*)
Graphite monochromator	*R*_int_ = 0.181
Detector resolution: 6.67 pixels mm^-1^	θ_max_ = 25.7°, θ_min_ = 1.6°
rotation method scans	*h* = −4→4
Absorption correction: integration (*X-RED32*; Stoe & Cie, 2002)	*k* = −18→19
*T*_min_ = 0.600, *T*_max_ = 0.976	*l* = −22→28
7609 measured reflections	

### Refinement

**Table:**

Refinement on *F*^2^	Primary atom site location: structure-invariant direct methods
Least-squares matrix: full	Secondary atom site location: difference Fourier map
*R*[*F*^2^ > 2σ(*F*^2^)] = 0.095	Hydrogen site location: inferred from neighbouring sites
*wR*(*F*^2^) = 0.229	H-atom parameters constrained
*S* = 0.97	*w* = 1/[σ^2^(*F*_o_^2^) + (0.0591*P*)^2^] where *P* = (*F*_o_^2^ + 2*F*_c_^2^)/3
2538 reflections	(Δ/σ)_max_ < 0.001
199 parameters	Δρ_max_ = 0.23 e Å^−^^3^
0 restraints	Δρ_min_ = −0.25 e Å^−^^3^

### Special details

**Table:**

Experimental. 215 frames, detector distance = 130 mm
Geometry. All e.s.d.'s (except the e.s.d. in the dihedral angle between two l.s. planes) are estimated using the full covariance matrix. The cell e.s.d.'s are taken into account individually in the estimation of e.s.d.'s in distances, angles and torsion angles; correlations between e.s.d.'s in cell parameters are only used when they are defined by crystal symmetry. An approximate (isotropic) treatment of cell e.s.d.'s is used for estimating e.s.d.'s involving l.s. planes.
Refinement. Refinement of *F*^2^ against ALL reflections. The weighted *R*-factor *wR* and goodness of fit *S* are based on *F*^2^, conventional *R*-factors *R* are based on *F*, with *F* set to zero for negative *F*^2^. The threshold expression of *F*^2^ > σ(*F*^2^) is used only for calculating *R*-factors(gt) *etc*. and is not relevant to the choice of reflections for refinement. *R*-factors based on *F*^2^ are statistically about twice as large as those based on *F*, and *R*- factors based on ALL data will be even larger.

### Fractional atomic coordinates and isotropic or equivalent isotropic displacement parameters (Å^2^)

**Table:**

	*x*	*y*	*z*	*U*_iso_*/*U*_eq_	
O3	0.7299 (11)	0.2631 (3)	0.1412 (2)	0.0806 (15)	
H2	0.7392	0.2252	0.1656	0.121*	
O2	0.0339 (12)	0.0664 (4)	−0.0715 (2)	0.0844 (15)	
N2	0.6571 (12)	0.1125 (4)	0.1845 (2)	0.0592 (14)	
N1	0.1412 (12)	0.1407 (4)	−0.0677 (2)	0.0635 (15)	
C13	0.6662 (13)	0.0823 (4)	0.2872 (3)	0.0533 (16)	
O1	0.1280 (16)	0.1866 (4)	−0.1100 (3)	0.110 (2)	
C1	0.4754 (13)	0.1458 (4)	0.0866 (3)	0.0536 (16)	
C7	0.5162 (13)	0.0880 (4)	0.1361 (3)	0.0579 (17)	
H7	0.4371	0.0321	0.1321	0.070*	
C10	0.8803 (16)	−0.0853 (5)	0.2670 (3)	0.0689 (19)	
H10	0.9492	−0.1411	0.2600	0.083*	
C11	0.8347 (15)	−0.0593 (5)	0.3228 (3)	0.0677 (18)	
H11	0.8708	−0.0967	0.3537	0.081*	
C5	0.2981 (13)	0.1707 (4)	−0.0126 (3)	0.0559 (16)	
C6	0.3326 (13)	0.1173 (4)	0.0339 (3)	0.0564 (16)	
H6	0.2586	0.0610	0.0299	0.068*	
C12	0.7342 (14)	0.0238 (5)	0.3314 (3)	0.0610 (18)	
O4	0.8401 (14)	0.1172 (4)	0.4082 (2)	0.1012 (18)	
N3	0.7006 (16)	0.0501 (5)	0.3918 (3)	0.0765 (17)	
C4	0.4026 (15)	0.2570 (5)	−0.0082 (3)	0.068 (2)	
H4	0.3767	0.2933	−0.0400	0.082*	
C2	0.5843 (14)	0.2325 (5)	0.0912 (3)	0.0608 (18)	
C9	0.8248 (14)	−0.0294 (5)	0.2215 (3)	0.0643 (18)	
H9	0.8636	−0.0472	0.1840	0.077*	
C3	0.5415 (16)	0.2853 (5)	0.0436 (3)	0.0676 (19)	
H3	0.6103	0.3421	0.0472	0.081*	
O5	0.5514 (16)	0.0028 (5)	0.4243 (3)	0.114 (2)	
C8	0.7111 (12)	0.0532 (4)	0.2312 (3)	0.0535 (16)	
C14	0.5391 (16)	0.1734 (4)	0.2969 (3)	0.0726 (19)	
H14A	0.5093	0.2023	0.2604	0.109*	
H14B	0.7128	0.2029	0.3216	0.109*	
H14C	0.3153	0.1720	0.3149	0.109*	

### Atomic displacement parameters (Å^2^)

**Table:**

	*U*^11^	*U*^22^	*U*^33^	*U*^12^	*U*^13^	*U*^23^
O3	0.091 (3)	0.065 (4)	0.083 (4)	−0.017 (2)	−0.011 (3)	−0.012 (3)
O2	0.107 (3)	0.066 (4)	0.079 (4)	−0.024 (3)	−0.002 (3)	−0.005 (3)
N2	0.056 (3)	0.057 (4)	0.064 (4)	−0.002 (2)	0.003 (3)	−0.007 (3)
N1	0.074 (3)	0.059 (5)	0.058 (4)	−0.002 (3)	0.005 (3)	0.003 (3)
C13	0.051 (3)	0.049 (4)	0.060 (4)	0.001 (3)	0.001 (3)	−0.007 (3)
O1	0.165 (5)	0.086 (5)	0.075 (4)	−0.011 (4)	−0.013 (3)	0.018 (3)
C1	0.049 (3)	0.049 (5)	0.062 (4)	−0.005 (3)	−0.001 (3)	−0.001 (3)
C7	0.051 (3)	0.053 (5)	0.069 (5)	0.000 (3)	0.002 (3)	−0.005 (4)
C10	0.078 (4)	0.051 (5)	0.076 (5)	0.007 (3)	−0.005 (4)	−0.002 (4)
C11	0.076 (4)	0.055 (5)	0.071 (5)	0.004 (3)	−0.005 (3)	0.003 (4)
C5	0.052 (3)	0.051 (5)	0.064 (5)	0.001 (3)	−0.002 (3)	−0.006 (4)
C6	0.056 (3)	0.047 (4)	0.066 (5)	−0.006 (3)	0.001 (3)	−0.001 (4)
C12	0.055 (3)	0.063 (5)	0.064 (5)	−0.002 (3)	−0.006 (3)	−0.007 (4)
O4	0.115 (4)	0.089 (5)	0.099 (4)	−0.007 (3)	−0.004 (3)	−0.027 (3)
N3	0.083 (4)	0.071 (5)	0.074 (5)	0.010 (3)	−0.005 (3)	−0.002 (4)
C4	0.061 (4)	0.054 (5)	0.090 (6)	0.003 (3)	0.002 (4)	0.003 (4)
C2	0.056 (3)	0.063 (5)	0.062 (5)	−0.003 (3)	−0.005 (3)	−0.008 (4)
C9	0.058 (3)	0.061 (5)	0.073 (5)	0.003 (3)	−0.003 (3)	−0.015 (4)
C3	0.076 (4)	0.054 (5)	0.072 (5)	−0.005 (3)	0.002 (4)	0.000 (4)
O5	0.140 (5)	0.112 (6)	0.093 (5)	−0.012 (4)	0.027 (4)	0.023 (4)
C8	0.040 (3)	0.060 (5)	0.059 (5)	−0.005 (3)	−0.006 (3)	−0.001 (4)
C14	0.077 (4)	0.060 (5)	0.081 (5)	0.015 (3)	0.001 (3)	−0.011 (4)

### Geometric parameters (Å, º)

**Table:**

O3—C2	1.339 (7)	C11—C12	1.375 (9)
O3—H2	0.8200	C11—H11	0.9300
O2—N1	1.235 (7)	C5—C6	1.364 (9)
N2—C7	1.269 (7)	C5—C4	1.412 (9)
N2—C8	1.430 (8)	C6—H6	0.9300
N1—O1	1.214 (7)	C12—N3	1.469 (9)
N1—C5	1.451 (8)	O4—N3	1.228 (8)
C13—C12	1.388 (9)	N3—O5	1.217 (8)
C13—C8	1.395 (8)	C4—C3	1.353 (9)
C13—C14	1.528 (9)	C4—H4	0.9300
C1—C6	1.377 (8)	C2—C3	1.382 (9)
C1—C2	1.423 (9)	C9—C8	1.388 (9)
C1—C7	1.462 (9)	C9—H9	0.9300
C7—H7	0.9300	C3—H3	0.9300
C10—C11	1.374 (9)	C14—H14A	0.9600
C10—C9	1.377 (9)	C14—H14B	0.9600
C10—H10	0.9300	C14—H14C	0.9600
			
C2—O3—H2	109.5	C11—C12—N3	116.4 (6)
C7—N2—C8	120.2 (6)	C13—C12—N3	119.5 (7)
O1—N1—O2	120.5 (6)	O5—N3—O4	122.4 (8)
O1—N1—C5	120.7 (7)	O5—N3—C12	119.0 (7)
O2—N1—C5	118.8 (6)	O4—N3—C12	118.5 (7)
C12—C13—C8	116.2 (6)	C3—C4—C5	118.0 (7)
C12—C13—C14	123.7 (6)	C3—C4—H4	121.0
C8—C13—C14	120.0 (6)	C5—C4—H4	121.0
C6—C1—C2	118.1 (6)	O3—C2—C3	119.7 (7)
C6—C1—C7	120.7 (6)	O3—C2—C1	120.6 (6)
C2—C1—C7	121.2 (6)	C3—C2—C1	119.7 (6)
N2—C7—C1	121.6 (6)	C10—C9—C8	120.3 (7)
N2—C7—H7	119.2	C10—C9—H9	119.8
C1—C7—H7	119.2	C8—C9—H9	119.8
C11—C10—C9	120.5 (7)	C4—C3—C2	122.0 (7)
C11—C10—H10	119.7	C4—C3—H3	119.0
C9—C10—H10	119.7	C2—C3—H3	119.0
C10—C11—C12	118.0 (7)	C9—C8—C13	120.8 (6)
C10—C11—H11	121.0	C9—C8—N2	121.1 (6)
C12—C11—H11	121.0	C13—C8—N2	118.0 (6)
C6—C5—C4	121.3 (6)	C13—C14—H14A	109.5
C6—C5—N1	120.5 (6)	C13—C14—H14B	109.5
C4—C5—N1	118.1 (6)	H14A—C14—H14B	109.5
C5—C6—C1	120.9 (6)	C13—C14—H14C	109.5
C5—C6—H6	119.6	H14A—C14—H14C	109.5
C1—C6—H6	119.6	H14B—C14—H14C	109.5
C11—C12—C13	124.0 (7)		
			
C8—N2—C7—C1	−176.5 (5)	C11—C12—N3—O4	132.5 (6)
C6—C1—C7—N2	177.9 (5)	C13—C12—N3—O4	−47.3 (8)
C2—C1—C7—N2	−1.6 (8)	C6—C5—C4—C3	0.5 (8)
C9—C10—C11—C12	−0.3 (9)	N1—C5—C4—C3	178.6 (5)
O1—N1—C5—C6	−176.0 (6)	C6—C1—C2—O3	−179.0 (5)
O2—N1—C5—C6	1.3 (8)	C7—C1—C2—O3	0.5 (8)
O1—N1—C5—C4	6.0 (8)	C6—C1—C2—C3	0.6 (8)
O2—N1—C5—C4	−176.8 (5)	C7—C1—C2—C3	−179.9 (5)
C4—C5—C6—C1	−0.9 (9)	C11—C10—C9—C8	−2.0 (9)
N1—C5—C6—C1	−178.9 (5)	C5—C4—C3—C2	0.4 (9)
C2—C1—C6—C5	0.3 (8)	O3—C2—C3—C4	178.6 (6)
C7—C1—C6—C5	−179.2 (5)	C1—C2—C3—C4	−0.9 (9)
C10—C11—C12—C13	1.9 (9)	C10—C9—C8—C13	2.8 (8)
C10—C11—C12—N3	−177.9 (5)	C10—C9—C8—N2	179.1 (5)
C8—C13—C12—C11	−1.1 (8)	C12—C13—C8—C9	−1.3 (7)
C14—C13—C12—C11	177.3 (6)	C14—C13—C8—C9	−179.7 (5)
C8—C13—C12—N3	178.7 (5)	C12—C13—C8—N2	−177.6 (5)
C14—C13—C12—N3	−3.0 (8)	C14—C13—C8—N2	3.9 (7)
C11—C12—N3—O5	−44.0 (8)	C7—N2—C8—C9	39.8 (7)
C13—C12—N3—O5	136.2 (6)	C7—N2—C8—C13	−143.9 (5)

### Hydrogen-bond geometry (Å, º)

**Table:**

*D*—H···*A*	*D*—H	H···*A*	*D*···*A*	*D*—H···*A*
O3—H2···N2	0.82	1.85	2.589 (8)	149
C6—H6···O2^i^	0.93	2.50	3.333 (9)	149
C4—H4···O4^ii^	0.93	2.59	3.274 (9)	131

Symmetry codes: (i) −*x*, −*y*, −*z*; (ii) *x*, −*y*+1/2, *z*−1/2.
